# Malignant Versus Benign Tumors of the Sinonasal Cavity: A Case-Control Study on Occupational Etiology

**DOI:** 10.3390/ijerph15122887

**Published:** 2018-12-17

**Authors:** Enzo Emanuelli, Vera Comiati, Diego Cazzador, Gloria Schiavo, Enrico Alexandre, Ugo Fedeli, Giuliana Frasson, Alessia Zanon, Alessandro Martini, Maria Luisa Scapellato, Giuseppe Mastrangelo

**Affiliations:** 1 Department of Neurosciences, Operative Unit of Otorhinolaryngology, University of Padova, Via Giustiniani 2, 35125 Padova, Italy; enzoemanuelli@libero.it (E.E.); minirosa@hotmail.it (G.S.); alexandre.enrico@gmail.com (E.A.); giuliana.frasson@hotmail.it (G.F.); alessiazanon@hotmail.it (A.Z.); alessandromartini@unipd.it (A.M.); 2 Department of Cardiac Thoracic Vascular Sciences and Public Health, Preventive Medicine and Risk Assessment Unit, University of Padova, Via Giustiniani 2, 35128 Padova, Italy; veracomiati@gmail.com (V.C.); marialuisa.scapellato@unipd.it (M.L.S.); giuseppe.mastrangelo@unipd.it (G.M.); 3 Epidemiological Department, Veneto Region. Passaggio Gaudenzio 1, 35131 Padova, Italy; ugo.fedeli@azero.veneto.it (U.F.)

**Keywords:** wood industry, leather industry, textile industry, metal transformation industry, sinonasal tumors, sinonasal adenocarcinoma, sinonasal squamous cell carcinoma, sinonasal inverted papilloma, occupational risk, case-control study

## Abstract

Case-control studies on malignant sinonasal tumors and occupational risk factors are generally weakened by non-occupational confounders and the selection of suitable controls. This study aimed to confirm the association between sinonasal malignant tumors and patients’ occupations with consideration for sinonasal inverted papillomas (SNIPs) as a control group. Thirty-two patients affected by adenocarcinoma (ADC) and 21 non-adenocarcinoma epithelial tumors (NAETs) were compared to 65 patients diagnosed with SNIPs. All patients were recruited in the same clinical setting between 2004 and 2016. A questionnaire was used to collect information on non-occupational factors (age, sex, smoking, allergies, and chronic sinusitis) and occupations (wood- and leather-related occupations, textile industry, metal working). Odds ratios (OR) with 95% confidence intervals (CI) associated with selected occupations were obtained by a multinomial and exact logistic regression. Between the three groups of patients, SNIP patients were significantly younger than ADC patients (*p* = 0.026). The risk of NAET increased in woodworkers (OR = 9.42; CI = 1.94–45.6) and metal workers (OR = 5.65; CI = 1.12–28.6). The risk of ADC increased in wood (OR = 86.3; CI = 15.2–488) and leather workers (OR = 119.4; CI = 11.3–1258). On the exact logistic regression, the OR associated to the textile industry was 9.32 (95%CI = 1.10–Inf) for ADC, and 7.21 (95%CI = 0.55–Inf) for NAET. Comparing sinonasal malignant tumors with controls recruited from the same clinical setting allowed demonstrating an increased risk associated with multiple occupations. Well-matched samples of cases and controls reduced the confounding bias and increased the strength of the association.

## 1. Introduction

### 1.1. Background

Sinonasal cancers are rare neoplasms (accounting for about 3% of all head and neck tumors) that originate mainly from the epithelial layer of the nose and paranasal sinuses [1]. The International Agency for Research on Cancer (IARC) classified occupational exposure to wood dust, leather dust, nickel compounds, isopropyl alcohol production, radium 226 and 228, and their relative products of decay as Group 1 carcinogens for the sinonasal mucosa [2]. A recent meta-analysis confirmed that exposure to wood and leather dust was strongly associated with sinonasal cancer. The greatest relative risk (RR) was calculated for adenocarcinoma (ADC). The corresponding pooled RR was 29.43 with a 95% confidence interval (95%CI) of 16.46–52.61 for wood dust, and 35.26 (95%CI: 20.62–60.28) for leather dust [3].

In a recent monocentric case-case study, ADCs were compared with non-adenocarcinoma epithelial tumors (NAETs) in patients recruited in the same clinical setting (Padua University Hospital), using questionnaires to collect information on occupation-related predictors [4]. Despite the limited sample size, that study confirmed the role of the traditional risk factors. The patients with ADC and NAET were found to be comparable in terms of their geographical background, social class, and symptoms, and they largely shared the same non-occupational risk factors (smoking, age, allergy, sinusitis). This reduced the effect of any confounders, increasing the validity and specificity of the association between sinonasal tumor and occupational exposures [5].

The different rates of NAET patients exposed to occupational risk factors reported in the literature (from about 20% to 8%) [4,6] may underestimate the true risk of developing ADC when cases of NAET are taken as a reference because the NAET carry their own risk of occupational exposure.

Sinonasal inverted papillomas (SNIP) are benign sinonasal tumors of unknown etiology, requiring surgical treatment [7]. A recent study [8] on 127 cases of SNIP and 337 hospital controls investigated the occupational risk factors for this benign tumor using questionnaires to ascertain the exposure to 17 occupational hazards. Exposure to welding fumes and organic solvents increased the odds ratio (OR) of developing a SNIP after adjusting for age, sex, area of residence, smoking and co-exposures. The risk tended to increase significantly with a greater continuous cumulative exposure and ordered cumulative exposure, but only for organic solvents. No other occupational hazards (particularly concerning wood, leather or textile dust) were associated with the risk of SNIP [8].

### 1.2. Objectives

The aim of the present study was to confirm the previously reported associations between sinonasal cancer and wood- and/or leather-related occupations, using a previously investigated case series of ADC and NAET [4] and adopting as a new reference patient with SNIP.

## 2. Materials and Methods

### 2.1. Setting and Participants

All patients underwent surgery at the Department of Otorhinolaryngology (Padua University Hospital) between 2004 and 2015 and were identified from the Department’s clinical records, which included patients’ demographic data, contact details and histological diagnoses. Patients with mesenchymal cancers and other neoplasms not arising from the sinonasal mucosa (e.g., melanoma) were excluded. All other patients were invited by phone to take part in the study. Written informed consent was obtained before proceeding. For elderly/disabled patients and those who had died, a letter requesting informed consent was sent to the patients’ homes, with a return addressed envelope, where a closest next of kin signed the informed consent. Three study groups were formed on the basis of patients’ histologies, i.e., patients with ADC, NAET, and SNIP.

### 2.2. Study Design

The three patient groups (diagnosed with ADC, NAET and SNIP) were compared pairwise in two case-control studies:

1. patients with ADC (cases) versus those with SNIP (controls);
2. patients with NAET (cases) versus those with SNIP (controls).

### 2.3. Variables

All information was collected by means of interview-based questionnaires administered by the same trained interviewer. Each patient’s occupational history was recorded using four dichotomous variables that encoded employment (yes/no) in one of the following industrial sectors:

- woodworking and cabinet-making (“woodworking” in the tables);
- footwear manufacturing and/or tanning (“leatherworking”);
- metal welding or galvanoplasty (“metalworking”);
- textiles (“textile manufacturing”).

Eight patients had been employed in more than one sector (6 from the ADC group, 1 from the NAET group; and 1 from the SNIP group). None of the patients had worked in the chemical industry (involving chromium, in particular) or in forestry, or other occupations associated with an increased risk of sinonasal cancer.

The potentially confounding non-occupational risk factors were encoded as follows: Age group at diagnosis (<55; 55–69; ≥70 years); gender; clinical history of allergies and chronic polypoid or non-polypoid sinusitis (as dichotomous variables); pack-years (PY, i.e., cigarettes a day/20 × years of smoking), classified as 0, ≤20, or >20 PY; and smoking status, i.e., never a smoker; ex-smoker who has quit smoking >15 years before the interview; current smoker or former smoker who has quit more recently (<15 years before).

### 2.4. Statistical Methods

The distribution of occupational and non-occupational variables by histological diagnosis was analyzed with the chi-square test, setting the threshold of significance at 0.05.

The association between sinonasal tumors and occupational history was investigated with a multinomial logistic (MNL) regression, where the categorical dependent variable was given an arbitrary numerical value (0 for SNIP; 1 for NAET, and 2 for ADC). The regression model estimated the OR and its 95%CI, and the *p*-value, indicating how the risk of either ADC or NAET changed with a one-unit change in an independent variable by comparison with the risk for the reference group (always SNIP). To adjust for demographic variables, all MNL regression models included the patient’s age at diagnosis and gender.

### 2.5. Sensitivity Analysis

The fact that some patients had been employed in more than one sector (see above) could introduce uncertainty into the output of the mathematical model. In order to reduce such uncertainty, a parallel statistical analysis was conducted to increase the robustness of the results (sensitivity analysis). The latter was obtained by excluding patients with more than one type of employment. Since some frequencies were 0 as a result of this exclusion, exact logistic regression was used instead of the maximum-likelihood-based multinomial logistic regression technique in order to produce more accurate inference.

The statistical analysis was performed with STATA software, (StataCorp.2015. Stata Statistical Software: Release 14; StataCorp LP, College Station, TX, USA).

## 3. Results

### 3.1. Participants

Among 66 patients diagnosed with sinonasal cancer (ADC, squamous cell carcinoma, undifferentiated carcinoma, mucoepidermoid carcinoma and adenoid cystic carcinoma), 13 were unwilling or unable to participate in the study. The original 75 SNIP patients thus became 65 (after excluding 6 patients who had died, 3 patients who were severely disabled, and one who was too young and therefore could have experienced only a short period of exposure).

The resulting 118 patients were divided into three groups based on their histological findings:

- an ADC group comprising 32 patients with intestinal-type adenocarcinoma (94%) arising from the ethmoid sinus (100%);
- a NAET group with 21 patients who had squamous cell carcinoma (or in some cases, adenoid-cystic carcinoma, undifferentiated carcinoma, or mucoepidermoid carcinoma) affecting the maxillary sinuses (38%), nasal septum (33%), and ethmoid sinuses (29%);
- a SNIP group containing 65 patients with benign tumors arising from the maxillary sinus (43%), ethmoid sinus (40%), sphenoid sinus (5%), nasal septum (3%), frontal recess (8%), or middle turbinate (1%).

### 3.2. Descriptive Data

Table 1 shows the distribution of the occupational and non-occupational variables by histological diagnosis (ADC, NAET and SNIP). The chi-square test revealed important differences for woodworking and leatherworking, particularly when comparing ADC with SNIP. Few patients reported ever having worked in textile manufacturing (4 ADC, 2 NAET and 0 SNIP). As for metalworking, the differences between the groups were not significant. Cases were older than controls, particularly concerning the ADC patients (*p* = 0.026), while other non-occupational risk factors showed no statistical significance.

### 3.3. Outcome Data: Main Findings

Table 2 shows the results of the comparison between NAET and SNIP, and between ADC and SNIP in the upper and lower panels, respectively. Each panel shows the unadjusted and adjusted analysis. In the upper panel, the risk of NAET increased significantly in association with woodworking (adjusted OR 9.42, *p* = 0.005; unadjusted OR 5.81, *p* = 0.017), and metal working (adjusted OR 5.65, *p* = 0.036). An increased OR emerged for leatherworking, although not reaching statistical significance. In the lower panel there was a massive increase in the risk of ADC associated with woodworking (adjusted OR 86.3, *p* < 0.001; unadjusted OR 51.7, *p* < 0.001) and leatherworking (adjusted OR 119.4, *p* < 0.001; unadjusted OR 120.4, *p* < 0.001). Finally, an age ≥ 70 years raised the risk of ADC (OR 8.60, *p* = 0.012).

Textile manufacturing was not included in the MNL analysis because no SNIP patient reported working in this sector (see Table 1). Using an exact logistic regression, the gender- and age-adjusted OR was 9.32 (95%CI: 1.10–Inf; *p* = 0.040) for ADC, and 7.21 (95%CI: 0.55–Inf; *p* = 0.129) for NAET.

### 3.4. Outcome Data: Sensitivity Analysis

Table 3 shows the results of the exact logistic regression after excluding the 8 patients employed in more than one industrial sector. In the upper panel, the NAET risk significantly increased with woodworking (adjusted OR 9.25, *p* = 0.028), and was of borderline significance for leatherworking. In the lower panel, a marked increase in the ADC risk emerged for woodworking (adjusted OR 130.2, *p* < 0.001; unadjusted OR 63.0, *p* < 0.001), and leatherworking (adjusted OR 92.99, *p* < 0.001; unadjusted OR 104.0, *p* < 0.001). Having worked for a period of time in the metal transformation industry was not associated with a higher risk of ADC. Age never showed a significant influence.

## 4. Discussion

### 4.1. Key Results

This study investigated occupational risk factors in patients who underwent surgery for the resection of adenocarcinoma or non-adenocarcinoma epithelial tumor (cases), and in patients who underwent endoscopic surgery for the removal of sinonasal inverted papillomas (controls). Wood-related and leather-related occupations were associated with a statistically very significant and much higher risk of sinonasal ADC, whilst this association was weaker though still significant for textile manufacturing. The risk of sinonasal NAET rose for woodworkers and metalworkers. The role of the main occupational risk factors was therefore confirmed. 

### 4.2. Limitations

An obvious limitation of this study lies in the small number of subjects involved. Despite the small sample size affecting the present as well as almost all studies on rare neoplasms such as sinonasal tumors, multiple occupational exposures showed an increased risk of developing both ADC and NAET. Considering ADC, huge relative risks partially managed the problem of sample size, which in particular affected the comparison of NAET with SNIP. To this end, we applied the multinomial logistic regression (Table 2) and the exact logistic regression (sensitivity analysis, Table 3). In Table 3, the risk of NAET was still significantly increased for wood working (*p* = 0.028), whilst a trend towards significance was calculated for leatherworking, *p* = 0.062.

This shortcoming was attenuated, however, because the sinonasal cancer risk in workers involved in the occupations considered here is generally high, and because the non-occupational risk factors assessed (age, sex, smoking, allergies, and chronic sinusitis) were largely shared by both cases as well as controls (see Table 1).

The main drawback of the small sample size is that some specific circumstances of exposure were not investigated. In a previous paper on occupational exposure in sinonasal cancer [4], circumstances of past occupational exposure were collected by means of a questionnaire. For the wood industry we estimated: Intensity (calendar year of first exposure was considered as a surrogate of intensity because wood dust exposure reduced over time due to technological advances); frequency (workshift frequency of jobs entailing high dust exposure as cutting, sanding, planning and use of compressed air); duration of wood dust exposure (<15 and ≥15 years); wood type (softwood/mixed wood types, hardwoods); and the use of protective equipment (masks, presence of local exhaust ventilation). For the leather industry, jobs were identified with a high exposure to leather dust (cutting, polishing and sole preparation), as well as jobs with a high exposure to mastic and/or solvents (shoe assembling and gluing). However, the small overall sample size and the lack of a sufficient number of SNIP cases (4 in the wood industry and 1 in the leather working, Table 1) prevented us from defining sub-groups of patients based on the above variables. Therefore, it was decided that only the occupation (wood working, leather working, metal working and textile manufacturing) and not the circumstances of exposure would be investigated.

All controls in the present study belonged to the same nosological category, whereas hospital-based case-control studies usually choose controls from multiple diagnostic categories and often from several different hospital departments too. For instance, d’Errico and colleagues [8] compared SNIP patients (their cases) with a control group of patients hospitalized in ear, nose and throat (ENT) wards (9 patients with nasal fracture, 32 with chronic otitis, 8 with sialadenitis, 44 with dizziness, and 6 with other diseases), or orthopedic departments (237 bone fractures other than those involving the hand, forearm or foot). Patients with rare, severe diseases (cases) may converge in a same hospital ward as others with common, more mild health problems (controls). The latter are more likely to live locally, and the former to come from further away, often crossing regional boundaries. That is why d’Errico [8] selected cases and controls from several hospitals in the same region, so as to balance the participants’ areas of residence. On these premises, it might be better to compare cases and controls recruited in the same clinical setting because their treatment requires the same specialist skills. Two such samples are more likely to be similar in many respects, and consequently more comparable. Having well-matched samples could reduce confounding biases and strengthen the associations, making it easier to identify real risk factors [9].

Our NAET group included several histologies (adenoid cystic carcinoma, undifferentiated carcinoma and mucoepidermoid carcinoma), as well as squamous cell carcinoma (SCC). The latter fact might be seen as a weakness of the study but the small number of patients and the weak evidence of an association between these histologies and exposure to wood and leather dust [6] make it unlikely that their inclusion could have severely biased our results.

SNIPs might be linked in some way with an occupational exposure, so patients with SNIPs may not be an ideal choice as controls. An occupational exposure was conjectured as contributory factor based on two case-control studies. In 50 SNIP cases and 150 controls, Sham et al. [10] found an industrial exposure (construction, textile, printing, papermaking, and electronic industries) in 20% of SNIP patients and in 5% of control group (*p* < 0.001). An outdoor occupation was also associated with SNIP (*p* = 0.02). In 127 cases of SNIP and 337 hospital controls, d’Errico et al. [8] investigated the following specific occupational exposures: arsenic, wood dust, leather dust, nickel compounds, chromium VI and its salts, polycyclic aromatic hydrocarbons, welding fumes, oil mists, formaldehyde, flour, cocoa powder, textile dusts, silica, coal dust, paint mists, strong acid mists and organic solvent vapors. The risk of SNIP was significantly increased for those reported exposure to welding fumes and organic solvents, with a dose/response relationship observed only in organic solvents. No other occupational exposure, including wood dust, textile dust and leather dust, was associated with the risk of developing SNIP. In the present study, normal controls were not available. Because exposure in the wood, leather, or textile industry was uncommon among SNIP (4/65, 1/65, 0/65, respectively, as shown in Table 1), it is highly unlikely that data from normal controls could provide any evidence that the wood, leather, or textile industry might act as risk factors in the development of SNIP. Therefore, both the published evidence and data from the present study demonstrate that ADC, NAET and SNIP do not share the same occupational risk factors.

Eight patients in our sample had been employed in several industrial sectors but the sensitivity analysis conducted after removing them confirmed the main results.

Given the above, the main limitations of the present study remain the use of SNIP patients as controls and the lack of analyses on specific circumstances of exposure, namely the length of the work in leather and wood-related jobs.

## 5. Generalizability

While it is well accepted that exposure to wood dust raises the risk of sinonasal ADC, it is still not clear as to what extent it increases the risk of SCC. Smaller excesses of sinonasal SCC have been observed [11,12,13,14,15,16] and some studies reported no such excess risk [17,18,19,20]. In two population-based case-control studies conducted in Washington State in 1979–1987, the ORs associated with having worked at some time in one of the four wood-related occupations were 2.4 (95%CI 0.8–6.7) for SCC of the sinonasal cavity [15]. Most of these studies on this issue lacked enough statistical power, however, because of the small number of cases (particularly of SCC). In a review of 12 case-control studies conducted in seven countries (involving a total of 680 male and 250 female cases, and 2349 male and 787 female controls), no association emerged for males between SCC and employment in any wood-related occupations (OR = 0.8 95%CI = 0.6–1.1), nor were the risks high for any job categories [21]. In another case-control study on 207 patients with sinonasal tumors (59 SCCs) and 409 controls conducted in France in 1986–1988, the ORs of SCC after exposure (for at least 6 months) to hardwood and softwood were 1.4 and 1.65, respectively (i.e., not significant) [22]. The estimated ORs from case-control studies for specific histological types of sinonasal cancer were included in a recent meta-analysis, which revealed that exposure to wood dust showed a much stronger association with ADC than with SCC of the sinonasal cavity [23]. Likewise, in the present study the adjusted OR in woodworkers was 86.3 (95%CI 15.2–488.0 and *p* < 0.001) for ADC, and 9.42 (95%CI 1.94–45.6 and *p* = 0.005) for NAET.

The aforementioned meta-analysis revealed a strong association between leather-related occupations and ADC. Two studies also reported data on SCC: The OR was 5.00 (0.44, 56.80) in a case-control study conducted in Piedmont (Italy) [24], and 1.50 (0.70, 3.00) in a pooled analysis of eight European case-control studies [25]. Accordingly, the age- and sex-adjusted OR for leather workers in the present study was 119.4 (95%CI 11.3–1258.0; *p* < 0.001) for ADC—the highest yet to be reported in the literature—and 7.92 (95%CI 0.64–97.6; *p* = 0.106) for NAET.

Data on the association between textile manufacturing and sinonasal cancer have been reported by industry, occupation, exposure, period of employment, histological type of cancer, and employee’s gender. Overall, the relative risks were much lower than in wood- or leather-related occupations [23]. Unlike wood or leather dust, chromium VI and nickel compounds, textile dust was not classified by the IARC as a human carcinogen (Group 1) causally related to sinonasal cancer [26]. An association emerges from the present results, however, with an adjusted OR of 9.32 for ADC (95%CI: 1.10–Inf; *p* = 0.040) and 7.21 for NAET (95%CI: 0.55–Inf; *p* = 0.129).

Welding and galvanoplasty were classified as “metalworking” in Table 1, Table 2 and Table 3. In case-control studies, the exposure to nickel and chromium (often concurrently) was mainly related to the welding of stainless steel, electrolytic metallurgy or spray painting. The associated levels of exposure were low, which this may explain the mainly negative results. Despite the latter fact, when Hernberg [13] studied the association between these activity-related exposures and sinonasal cancer, the OR was 2.7 (95%CI 1.1–6.6) for exposure to chromium, and 2.4 (95%CI 0.9–6.6) for exposure to nickel. In the present study, the adjusted ORs for metalworking were 3.58 (95%CI 0.38–33.2; *p* = 0.263) for ADC, and 5.65 (95%CI 1.12–28.6; *p* = 0.036) for NAET. Welding is one of the few jobs possibly associated with the development of SNIP. Therefore, the latter ORs might be underestimated.

## 6. Conclusions

Based on the consistency between our findings and those in literature, it seems reasonable to consider SNIP patients as valid controls in case-control studies on occupational risks associated with sinonasal malignancies. Because such cases (malignant tumors) and controls (benign tumors) could be found at the same source (ENT departments), the conduct of further studies on the etiology of sinonasal cancer can be facilitated.

## Figures and Tables

**Table 1:** Distribution of study variables among cases with adenocarcinoma (ADC, *n* = 32) or non-adenocarcinoma epithelial tumor (NAET, *n* = 21), and controls with sinonasal inverted papilloma (SNIP, *n* = 65).

Variables	Classes	ADC, *n* (%)	NAET, *n* (%)	SNIP, *n* (%)	*p* (Chi-square)
Sex	Males	26 (81%)	14 (67%)	49 (75%)	0.483
	Females	6 (19%)	7 (33%)	16 (25%)	
Age classes	<55 years	7 (22%)	6 (29%)	23 (35%)	0.026
	55–69 years	8 (25%)	8 (38%)	29 (45%)	
	≥70 years	17 (53%)	7 (33%)	13 (20%)	
Smoking habits	Never	17 (53%)	15 (71%)	25 (39%)	0.099
	Former	8 (25%)	2 (10%)	21 (32%)	
	Current	7 (22%)	4 (19%)	19 (29%)	
Packyears	0	17 (53%)	15 (71%)	25 (38%)	0.067
	0.1–20	7 (22%)	4 (19%)	17 (26%)	
	>20	8 (25%)	2 (10%)	23 (35%)	
History of sinusitis/allergy	No	22 (69%)	15 (71%)	36 (55%)	0.271
	Yes	10 (31%)	6 (29%)	29 (45%)	
Wood working	Never	15 (47%)	16 (76%)	61 (94%)	<0.001
	Ever	17 (53%)	5 (24%)	4 (6%)	
Leather working	Never	21 (66%)	19 (90%)	64 (98%)	<0.001
	Ever	11 (34%)	2 (10%)	1 (2%)	
Metal working	Never	29 (91%)	17 (81%)	61 (94%)	0.210
	Ever	3 (9%)	4 (19%)	4 (6%)	
Textile manufacturing	Never	28 (88%)	19 (90%)	65 (100%)	0.018
	Ever	4 (12%)	2 (10%)	0 (0%)	

**Table 2:** Multinomial logistic regression estimating the odds ratio (OR) with a 95% confidence interval (95%CI) and two-tailed *p*-value (*p*) for a categorical dependent variable taking the value 0 for sinonasal inverted papilloma (SNIP), 1 for non-adenocarcinoma epithelial tumors (NAET) and 2 for adenocarcinoma (ADC). Unadjusted and age–sex adjusted risk estimates in each panel.

Variables	OR (95%CI)	*p*	OR (95%CI)	*p*
**NAET versus SNIP**				
	Unadjusted Analysis		Adjusted Analysis	
Sex ^#^			2.19 (0.65; 7.34)	0.205
Age: 55-69 years ^§^			1.21 (0.32; 4.55)	0.778
Age: ≥70 years ^§^			2.53 (0.61; 10.4)	0.200
Wood working °	5.81 (1.36; 24.8)	0.017	9.42 (1.94; 45.6)	0.005
Leather working °	8.43 (0. 71; 100)	0.092	7.92 (0.64; 97.6)	0.106
Metal working °	3.00 (0.59; 15.3)	0.188	5.65 (1.12; 28.6)	0.036
**ADC versus SNIP**				
	Unadjusted Analysis		Adjusted Analysis	
Sex ^#^			1.27 (0.24; 6.26)	0.775
Age: 55–69 years ^§^			1.27 (0.24; 6.68)	0.775
Age: ≥70 years ^§^			8.60 (1.60; 46.1)	0.012
Wood Working °	51.7 (12.2; 219)	<0.001	86.3 (15.2; 488)	<0.001
Leather Working °	120.4 (12.6; 1147)	<0.001	119.4 (11.3; 1258)	<0.001
Metal Working °	2.54 (0.31; 21.1)	0.388	3.58 (0.38; 33.2)	0.263

^#^ Reference: females. ^§^ Reference: patients aged <55 years. ° Reference: patients never employed in the index industrial sector.

**Table 3:** Exact logistic regression estimating the odds ratio (OR) with a 95% confidence interval (95%CI) and two-tailed *p*-value (*p*) for a case-control study.

Variables	OR (95%CI)	*p*	OR (95%CI)	*p*
**NAET versus SNIP**				
	Unadjusted Analysis		Adjusted Analysis	
Sex ^#^			2.53 (0.59; 10.96)	0.249
Age ^§^			1.33 (0.72; 2.43)	0.386
Wood working °	6.66 (0.98; 52.7)	0.053	9.25 (1.24; 81.60)	0.028
Leather working °	11.3 ^†^ (0.86; +Inf)	0.065	12.22 ^†^ (0.88; +Inf)	0.062
Metal working °	3.79 (0.49; 26.1)	0.232	4.65 (0.56; 35.4)	0.173
**ADC versus SNIP**				
	Unadjusted Analysis		Adjusted Analysis	
Sex ^#^			3.44 (0.26; 54.7)	0.4785
Age ^§^			2.25 (0.97; 5.68)	0.0598
Wood Working °	63.0 (12.0; 494)	0.000	130.2 (15.9; 2311)	0.0000
Leather Working °	104.0 ^†^ (13.3; +Inf)	0.000	92.99 ^†^ (10.9; +Inf)	0.0000
Metal Working °	2.93 ^†^ (0; 29.2)	1.0000	4.01 ^†^ (0; 46.7)	1.0000

In the upper panel, cases = non-adenocarcinoma epithelial tumors (NAET) and controls = sinonasal inverted papilloma (SNIP). In the lower panel, cases = adenocarcinoma (ADC) and controls = sinonasal inverted papilloma (SNIP). Unadjusted and age–sex adjusted risk estimates are given in each panel. ^#^ Reference: females. ^§^ Reference: patients aged <55 years. ° Reference: patients never employed in the index industrial sector. ^†^ Median Unbiased Estimates.
